# Respiratory oscillometry and functional analyses in patients with idiopathic scoliosis

**DOI:** 10.1590/1414-431X2023e12898

**Published:** 2023-11-03

**Authors:** C.M.S. Sousa, A.L.C. Pessoa, L.E. Carelli, C.O. Ribeiro, A.J. Lopes, P.L. Melo

**Affiliations:** 1 Laboratório de Instrumentação Biomédica, Instituto de Biologia, Faculdade de Engenharia, Universidade do Estado do Rio de Janeiro, Rio de Janeiro, RJ, Brasil; 2 Hospital Universitário Pedro Ernesto, Universidade do Estado do Rio de Janeiro, Rio de Janeiro, RJ, Brasil; 3 Instituto Nacional de Traumatoortopedia, Rio de Janeiro, RJ, Brasil; 4 Laboratório de Função Pulmonar, Hospital Universitário Pedro Ernesto, Universidade do Estado do Rio de Janeiro, Rio de Janeiro, RJ, Brasil; 5 Laboratório de Pesquisa Clínica e Experimental em Biologia Vascular, Universidade do Estado do Rio de Janeiro, Rio de Janeiro, RJ, Brasil

**Keywords:** Respiratory oscillometry, Forced oscillation technique, Scoliosis, Functional capacity

## Abstract

Scoliosis is a condition that affects the spine and causes chest rotation and trunk distortion. Individuals with severe deformities may experience dyspnea on exertion and develop respiratory failure. Respiratory oscillometry is a simple and non-invasive method that provides detailed information on lung mechanics. This work aims to investigate the potential of oscillometry in the evaluation of respiratory mechanics in patients with scoliosis and its association with physical performance. We analyzed 32 volunteers in the control group and 32 in the scoliosis group. The volunteers underwent traditional pulmonary function tests, oscillometry, and the 6-minute walk test (6MWT). Oscillometric analysis showed increased values of resistance at 4 Hz (R4, P<0.01), 12 Hz (R12, P<0.0001), and 20 Hz (R20, P<0.01). Similar analysis showed reductions in dynamic compliance (Cdyn, P<0.001) and ventilation homogeneity, as evaluated by resonance frequency (fr, P<0.001) and reactance area (Ax, P<0.001). Respiratory work, described by the impedance modulus, also showed increased values (Z4, P<0.01). Functional capacity was reduced in the group with scoliosis (P<0.001). A significant direct correlation was found between Cobb angle and R12, AX, and Z4 (P=0.0237, P=0.0338, and P=0.0147, respectively), and an inverse correlation was found between Cdyn and Cobb angle (P=0.0190). These results provided new information on respiratory mechanics in scoliosis and are consistent with the involved pathophysiology, suggesting that oscillometry may improve lung function tests for patients with scoliosis.

## Introduction

Scoliosis is a disease that primarily affects the spine, causing rotation of the thorax and distortion of the trunk (1). It affects about 10% of the world's population with women predominating. In most cases, it is diagnosed at puberty and may present as idiopathic scoliosis and be asymptomatic until middle age, when individuals with severe deformities may experience dyspnea and develop respiratory failure on exertion or, less frequently, heart failure (1).

Due to changes related to scoliosis, many patients may have limitations in performing exercises. Some authors attribute exercise intolerance to the presence of physical deconditioning or reduction in lean mass, which is usually present in these patients. The six-minute walk test (6MWT) is an easy-to-apply test that allows the assessment and classification of the functional capacity of exercise in patients with obstructive and resistive diseases, being also a good predictor of mortality in patients with chronic obstructive pulmonary disease (2).

Individuals with scoliosis may have reduced lung volume as measured by lung function tests, with spirometry and whole-body plethysmography being the most commonly used. These alterations are attributed to changes in the thoracic cage resulting in abnormal lung and chest wall biomechanics and restrictive ventilatory disorders (3). However, both tests require the patient to understand, cooperate, and be able to perform inspiratory and expiratory maneuvers with maximum effort (4).

Respiratory oscillometry, also known as the forced oscillation technique, was described in 1954 by Dubois and collaborators as a non-invasive method for lung evaluation, and has been improved ever since (5-7). Currently, this technique has shown greater relevance in pulmonary function laboratories, as it provides new information on pulmonary mechanics, allowing a better understanding of the pathophysiology (5-7).

Measurements of chest wall recoil are seldom performed in clinical practice because it is very difficult to obtain reliable data in untrained subjects. Therefore, the ability of oscillometry in providing information on the mechanical behavior of airways, lungs, and chest wall is of special interest in the analysis of patients with scoliosis (8). Although this method has been successfully applied to improve our understanding of abnormal respiratory mechanics in several diseases (6), there are few data in the literature about the use of oscillometry in scoliosis. In fact, only one study has addressed this question (8). The cited study examined a small group of patients with scoliosis (n=7) without considering the effects of spinal inclination and functional level of these patients.

Based on the above considerations, the main objectives of the present study were: 1) to use oscillometry to improve our understanding of the changes in respiratory mechanics in idiopathic scoliosis; 2) to study the associations between spinal inclination and alterations observed by oscillometry and perform functional capacity evaluation. The working hypothesis proposed is that the presence of scoliosis can lead to changes in the resistive and reactive properties of the respiratory system.

## Material and Methods

The present work was developed at the Biomedical Instrumentation Laboratory of the State University of Rio de Janeiro. All analyses were conducted on the same day in this cross-sectional study, which was approved by the Ethics Committee of the Pedro Ernesto University Hospital (protocol 2927/2011) and registered with the Brazilian Clinical Research Platform (81943417.9.0000.5259). All volunteers signed an informed consent form, and the study was conducted according to the principles of the Declaration of Helsinki.

### Volunteers

A total of 64 volunteers were analyzed, 32 in the control group and 32 in the scoliosis group. The control group included individuals with normal spirometry and body mass index (BMI) within the normal range. To determine the scoliosis curvature angle, we used the Cobb method, which is calculated through panoramic radiography of the spine, as described in the literature (9). Following the definition of scoliosis (9), patients with a Cobb angle greater than 10° were accepted. In both groups, the exclusion criteria were previous lung disease and history of smoking or COVID-19.

### Traditional pulmonary function analysis

Spirometry analysis was performed according to the American Thoracic Society/European Respiratory Society standards (10,11). The analyzed parameters were forced expiratory volume in the first second (FEV1), forced vital capacity (FVC), FEV1/FVC ratio, and forced expiratory flow (FEF) between 25 and 75% of the FVC (FEF/FVC) ratio. These parameters were recorded as absolute values and as percentage of predicted values (% of predicted), and the reference values were obtained from the equations of Pereira et al. (12). Forced expiratory maneuvers were repeated until three sequential measurements were obtained. The studied indexes were obtained using the best curve, which was selected based on the higher values of FEV1 plus FVC. The software automatically detected non-acceptable maneuvers according to American Thoracic Society (ATS) criteria, providing quality control of spirometric exams.

Plethysmography analyses were conducted with a constant volume and variable pressure plethysmograph (HD CPL nSpire Health Ltd., UK). The evaluated parameters were total lung capacity (TLC) and residual volume (RV), as well as the RV/TLC relationship. Airway resistance (Raw) was also measured. The reference values were based on the equations described by Neder et al. (13).

### Respiratory oscillometry

These measurements were performed in the frequency range between 4 and 32 Hz using a previously described instrument (14) according to standard recommendations (7). To perform these exams, the volunteer remains in a sitting position, with the head in a neutral position, with a nose clip and compression of the cheeks by the individual himself with open hands to reduce the upper airway shunt. Three acceptable tests of 16 s were performed, and the mean score was recorded. As recommended (7), the coefficient of variation of respiratory resistance at the lowest frequency (4 Hz) for the used measurements was ≤10%. The test was considered adequate if the volunteers presented stable tidal volumes and rates and were free of pauses. Common artifacts such as swallows, coughs, and leaks were identified by evaluating flow and pressure signals, and the acquisition was repeated until three stable flows without artifacts were obtained. To reduce the influence of spontaneous breathing, only exams with a coherence function ≥0.9 in the whole studied frequency range were accepted. The oscillometric examination was performed before spirometry and plethysmography tests. This prevents altering the tone of bronchial muscles due to the maximum effort made in these maneuvers and its possible effects in oscillometric results.

Resistance represents all elements that oppose airflow (15,16). The resistance curves are interpreted using resistance at 4 Hz (R4), related to the total respiratory resistance, and at 12 Hz (R12) and 20 Hz (R20), which are linked to the most central airways. We also evaluated the frequency dependence of the resistance represented by the difference between R4 and R20 (R4-R20), associated with ventilation homogeneity (17,18).

The reactance curves are interpreted using dynamic compliance (Cdyn), which includes the effects of airways, lungs, and chest wall. The reactance at 4 Hz (X4) was used to calculate this parameter (Cdyn=1/2πfX4). When there is a cancelation of the effects of compliance and inertia, it occurs at the resonance frequency (Fr), which is linked to changes in the homogeneity of elastic properties (17,19). Reactance is also interpreted using the area under the reactance curve (Ax). This parameter was recently identified as suitable for predicting the prognosis of patients with chronic obstructive pulmonary disease (COPD) (20) and is defined as the area inscribed by the Xrs curve between the lowest measured frequency and Fr (6). The respiratory system's total mechanical load was studied by analyzing the 4 Hz impedance module (Z4), which integrates resistive and elastic respiratory load.

### Six-minute walk test

The 6MWT test was performed after the participant had rested for at least 10 min after the pulmonary function tests. The individual was instructed to walk for six minutes in a linear route of 30 meters. The volunteer's vital signs were measured (heart rate, blood pressure, and peripheral oximetry) at the beginning and the end of the test. The test was repeated twice at 10-min intervals, and only the second test was considered valid (21). The measurement of the distance covered in the exam was recorded, and the reference equation by Enright and Sherrill was used to estimate the predicted values (2,22).

### Statistical processing, presentation, and analysis

Results are reported as means±SD and described in tables and boxplots. Statistical analysis was performed using the ORIGIN 8.0 program (Microcal Software Inc., USA).

Initially, sample distribution was evaluated using the Shapiro-Wilk test. The independent or dependent *t*-test was used when the data showed a normal distribution, while a non-parametric test was used when the data did not show a normal distribution (Mann-Whitney or Wilcoxon). Differences with P≤0.05 were considered statistically significant. Spearman's correlation coefficient was used to assess the associations between the oscillometric parameters and Cobb angle.

The required sample size was calculated based on the dynamic compliances observed in controls and patients with scoliosis, which was obtained in a pilot study with a smaller number of patients (23). Using MedCalc^®^ software (MedCalc Software, Belgium) and assuming 10% type I and type II errors, the minimum sample size was 32 volunteers in each group.

## Results

Table 1 describes the biometric and spirometric results of the study groups. There was no difference between groups concerning biometric characteristics, while all of the spirometric parameters were significantly reduced in the scoliosis group.

**Table 1:** Biometric and spirometric results and the Cobb angle of the studied groups.

	Control (n=32)	Scoliosis (n=32)	P
Age (years)	23.15±2.89	24.87±9.42	ns
Weight (kg)	59.01±7.83	55.30±11.20	ns
Height (cm)	165.43±7.54	161.81±11.40	ns
BMI (kg/m^2^)	21.52±2.05	21.16±3.81	ns
Cobb angle (degrees)	-	53.47±26.00	-
Gender (F/M)	23/9	23/9	-
FEV1 (L)	3.60±0.82	2.42±0.85	<0.0001
FEV1 (%)	104.09±13.89	74.54±24.61	<0.0002
FVC (L)	4.06±0.90	3.00±1.00	0.0002
FVC (%)	101.69±13.41	80.61±26.35	0.0002
FEV1/FVC	89.68±5.48	80.68±7.67	<0.0001
FEV1/FVC (%)	102.06±5.09	92.74±9.45	<0.0001
FEF25-75 (%)	4.34±1.22	2.49±1.04	<0.0001

Data are reported as mean±SD except for gender, which is reported as absolute number. BMI: body mass index; FEV1: forced expiratory volume in one second; FVC: forced vital capacity; FEF25-75: difference of forced expiratory flow between 25 and 75% of FVC (mean forced expiratory flow); n: number of patients evaluated. P<0.05 was considered statistically significant (*t*-test). ns: not significant.

Table 2 presents the plethysmographic absolute and predicted values obtained for the scoliosis group. Significant differences were found in all parameters. An average (±SD) value of 2.9±1.8 (cmH_2_O^.^s^.^L^-1^) was observed for the Raw in 31 patients with scoliosis. One patient was not able to produce adequate technical maneuvers for Raw measurement.

**Table 2:** Predicted and measured plethysmographic values of the scoliosis group.

	Predicted value (n=32)	Scoliosis (n=32)	P
TLC (L)	5.09±1.11	4.56±1.23	<0.05
RV (L)	1.31±0.30	1.77±0.88	<0.01
RV/TLC	25.25±2.81	38.68±14.22	<0.0001

Data are reported as mean±SD. TLC: total lung capacity; RV: residual volume; RV/TLC: ratio between residual volume and total lung capacity; n: number of patients evaluated. P<0.05 was considered statistically significant (*t*-test).

**Figure 1:**
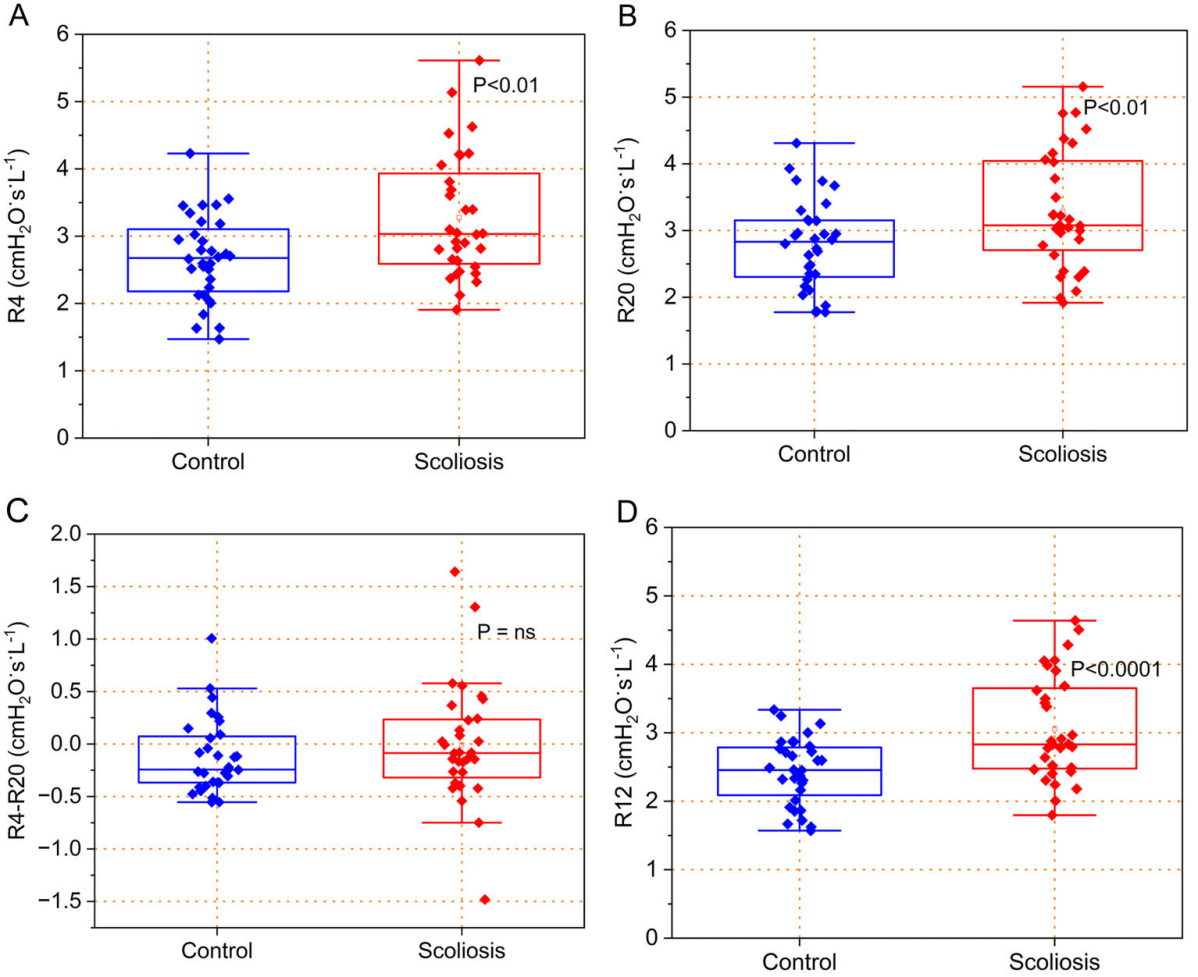
Effect of scoliosis on resistive parameters. **A**, R4, resistance at 4 Hz; **B**, R20, resistance at 20 Hz; **C**, R4-R20, frequency dependence of the resistance represented by the difference between R4 and R20; **D**, R12, resistance at 12 Hz. The top and bottom lines of the box plots are the 25th- to 75th-percentile values, while the circle represents the mean value, and the bar across the box represents the 50th-percentile value. The whiskers outside the box represent the 10th-to 90th-percentile values. Mann-Whitney test. ns: not significant.

Comparing the control and scoliosis groups, significant increases were observed at R4 (Figure 1A, P<0.01), R20 (Figure 1B, P<0.01), and R12 (Figure 1D, P<0.0001). No significant changes were observed in R4-R20 (Figure 1C, P=0.31).

**Figure 2:**
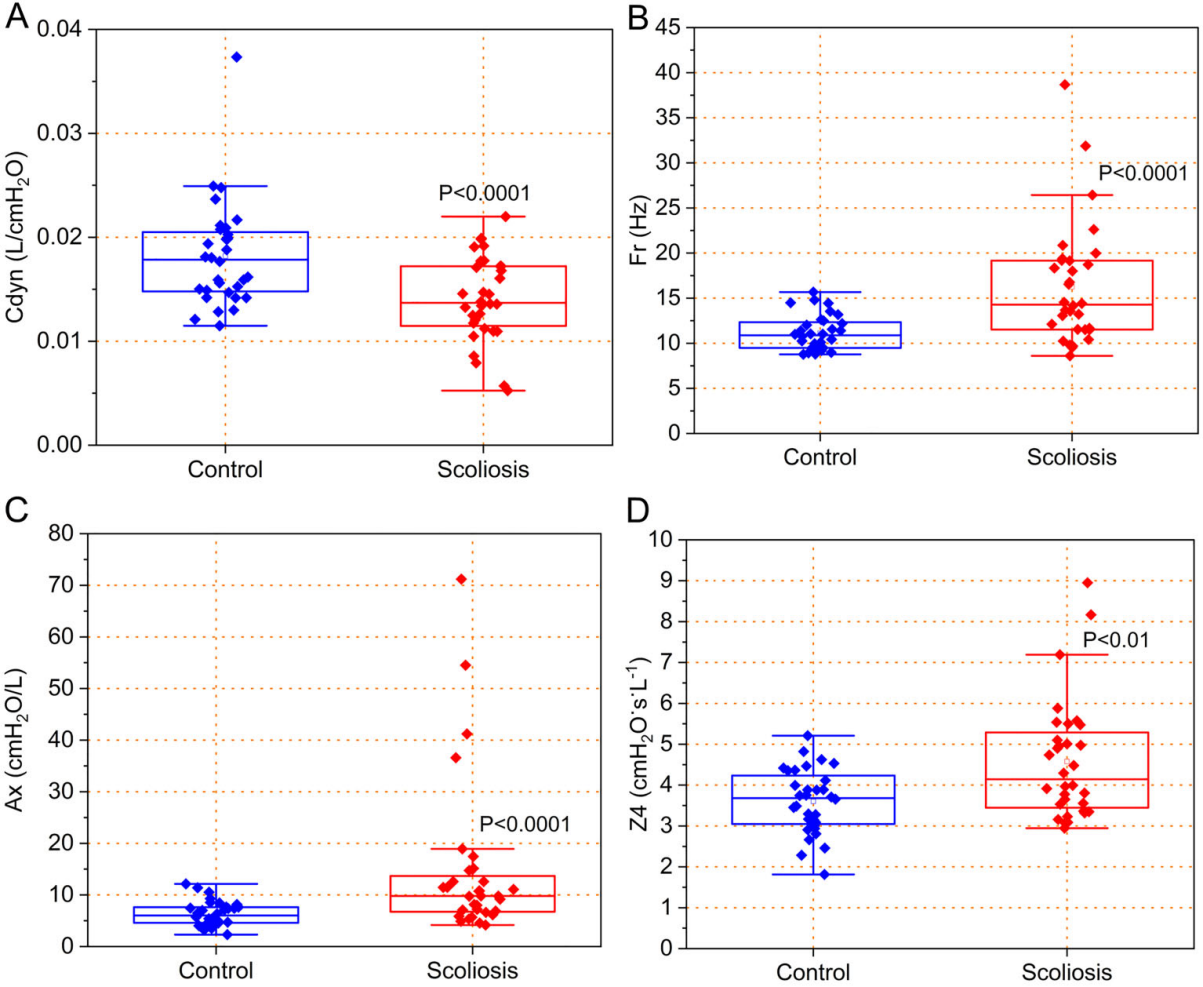
Effect of scoliosis on reactive parameters. **A**, Cdyn, dynamic compliance; **B**, fr, resonance frequency; **C**, Ax, reactance area; and **D**, Z4, impedance modulus at 4 Hz. The top and bottom lines of the box plots are the 25th- and 75th-percentile values while the circle represents the mean value, and the bar across the box represents the 50th-percentile value. The whiskers outside the box represent the 10th- to 90th-percentile values. Mann-Whitney test.

Considering the reactive parameters, similar comparisons showed significant alterations in Cdyn (Figure 2A, P<0.001), Fr (Figure 2B, P<0.001), Ax (Figure 2C, P<0.001), and Z4 (P<0.01). The interested reader may find in the Supplementary Material section a detailed description of the statistics used in previous comparisons (Supplementary Tables S1 to S4), as well as the correlations among oscillometric, spirometric (Supplementary Table S5), and plethysmographic (Supplementary Table S6) parameters.

A significant (P<0.0001) reduction of the 6MWT distance was found in the scoliosis group (535.29±110.43 m) compared with predicted values (725.35±86.02 m).

Table 3 presents the correlations between oscillometry and Cobb angle. We observed significant associations between R12, Cdyn, Ax, and Z4.

**Table 3:** Correlation coefficient (r), determination coefficient (r^2^), and correlation significance level (P) between oscillometric parameters and Cobb angle.

Cobb angle	R4	R20	R4-R20	R12	Cdyn	fr	Ax	Z4
R	0.2451	0.2515	-0.0715	0.3990	-0.4394	0.2853	0.3763	0.4274
r^2^	0.0601	0.0632	0.0051	0.1592	0.1931	0.0814	0.1416	0.1827
P	0.1764	0.1650	0.6972	**0.0237**	**0.019**	0.1135	**0.0338**	**0.0147**

Significant correlations are highlighted in bold. R4: resistance at 4 Hz; R20: resistance at 20 Hz; R4-R20: frequency dependence of resistance; R12: resistance at 12 Hz; Cdyn: dynamic compliance; fr: resonance frequency; Ax: area under the reactance curve; Z4: respiratory impedance module.

## Discussion

This study tested the hypothesis that scoliosis can introduce changes in the resistive and reactive properties of the respiratory system. Three major findings were obtained: 1) respiratory oscillometry provided a detailed description of the scoliosis pathophysiology, showing that this disease results not only in a reduction of respiratory compliance, but also in an increase in resistance and ventilation heterogeneity; 2) changes in oscillometric parameters were associated with the curvature of the spine; and 3) oscillometric parameters were also associated with the reduction in functional performance.

Table 1 shows that the studied groups were homogeneous, without significant biometric differences. Scoliosis affects the spine and distorts the trunk due to a lateral curvature of the spine, and its compensation restricts chest movement during ventilation. Patients in these conditions are expected to present restrictive respiratory disorders, with a reduction in total lung capacity and maintenance or increase in the FEV1/FVC ratio. The scoliosis group showed significant reductions in FEV_1_ (%), FEV_1_ (L), FVC (%), FVC (L), FEV1/FVC (L), FEV_1_/FVC (%), and FEF25-75%, which can be explained by extrinsic chest compression that restricts lung expansion (24,25).

Plethysmographic exams (Table 2) showed significant reductions in TLC. Spinal curvatures reduce tidal volume and, as a consequence, reduce total lung capacity (26). Air trapped in the lungs results in increased RV and RV/TLC ratio, indicating airway narrowing (27). This is consistent with the observed increase in RV and RV/TLC ratio.

Considering the Raw values in the scoliosis group, Pereira and Moreira (28) suggest classification values between 2.5 to 4.4 as mild severity, 4.5 to 8.0 as moderate, and above 8.0 as severe. Our sample included 10 patients that can be considered mild, 3 moderate, and 1 severe, while 17 patients scored below 2.5 cmH_2_O^.^s^.^L^-1^. The increased values can be attributed to extrinsic compression in the chest causing obstruction of airflow in the airways (29,30).

The increase in resistance values (Figure 1) are in line with the observed increase in Raw evaluated by body plethysmography. These findings are in line with those obtained by van Noord et al. (8), which attributed the increase to a marked decrease in pulmonary volume. A similar decrease in pulmonary volume was observed in the present study (Table 2). More specifically, the abnormal resistance values can be attributed to chest wall deformity due to curvature and abnormal rotation of the spine leading to lung deformation and restriction in chest expansion (29-33). In addition, the higher resistance may also be explained, at least in part, to an increase in tissue resistance.

The analysis of reactive parameters showed a significant reduction in Cdyn (Figure 2A). This result is in close agreement with the involved physiology and can be attributed to the complex effects of the abnormal curvature of the spine, restricting some lung areas and limiting or making lung expansion difficult due to changes in the biomechanics of the thorax (31,34,35). These elastic changes may also explain the elevations in fr (Figure 2B) and Ax (Figure 2C). These alterations also demonstrate abnormal changes in the ventilation homogeneity in the airways, indicating a higher level of obstruction or pulmonary restriction in these patients (6).

Z4 was significantly increased in the scoliosis group (Figure 2D), indicating increased work of breathing. Patients with scoliosis usually have a greater work of breathing because they must expend more energy to overcome the chest deformity and expand the lungs (31). These results are in line with the dyspnea usually reported by these patients.

The scoliosis group obtained 6MWT values significantly lower than expected by the reference equation (2). This can be attributed to exercise restriction due to a reduction in lean body mass and/or cardiovascular impairment due to spinal curvature (36,37).

Perhaps the most interesting results of the study are the correlations observed between oscillometry and Cobb angle (Table 3). R12 showed a significant and direct correlation, demonstrating that an increase in Cobb angle was related to increased resistance. Interestingly, R4 was not associated with Cobb angle. Resistance of the respiratory system measured by oscillometry describes frictional losses in airways, lung tissues, and chest wall. Resistance above 5 Hz indicates airway resistance, while tissue resistance becomes progressively more important as frequency decreases below 5 Hz (32). Thus, the findings indicated that the increase in Cobb angle was more closely related to alterations in the airways than in pulmonary and chest wall tissue. In addition, R20 and R4-R20 were not sufficiently sensitive to airway changes to be associated with Cobb angle.

Direct and significant associations were also observed with AX and Z4, which provided evidence that increases in Cobb angle affect ventilation heterogeneity and increase work of breathing. These findings were consistent with the typical reduction in lung and chest wall compliance shown by patients with scoliosis (2,36). This reduction was confirmed by the significant and inverse correlation between Cdyn and Cobb angle. This can be attributed to the restricted expansion of the ribcage in these patients due to changes in the curvature of the spine (25,38).

Respiratory oscillometry has been widely perceived as the state-of-the-art lung function analysis (39). Although it provides simple and detailed results that are particularly important in several clinical applications, this method is still not widely used because the parameters obtained are difficult to interpret by the untrained clinician (6) and because the method is still evolving (40). In order to improve the interpretation of oscillometric measurements, the associated parameters must be related to tangible and quantifiable clinical properties. The present study contributes in this direction by showing that the ventilatory changes evaluated by oscillometry are associated with the curvature of the spine in scoliosis.

A recent review (6) emphasized that, despite great promise as a useful clinical tool, more evidence of clinical utility is needed before oscillometry becomes routinely used for diagnosing or monitoring respiratory disease. It was also pointed out that clinical application of oscillometry is less established in restrictive lung disease than in obstructive lung disease. In this practical context, the present study's findings could help improve clinical practice showing that oscillometry may accurately detect restrictive abnormalities in scoliosis.

Although strict criteria were adopted in the design of this study, the findings are subject to three limitations. First, it was a single-center study; hence, the results may not represent the entire patient population. It could be argued that because only 64 subjects were recruited, the exact values remain unknown. Future studies should include a larger number of subjects. However, this preliminary analysis significantly contributes to an important debate in the literature concerning the use of oscillometry in restrictive lung diseases, particularly in patients with scoliosis. Secondly, volunteers with scoliosis could have been divided into groups with and without lung function abnormalities to assess the ability of oscillometry to diagnose these changes. Lastly, this study focused on whole-breath impedance measurements. Within-breath analysis was not evaluated. Such limitations can be taken as suggestions for future research.

In conclusion, this study improved our knowledge regarding respiratory abnormalities in patients with scoliosis by providing a detailed analysis of the changes in resistance and reactance in these patients. In close agreement with the working hypothesis, it was shown that scoliosis not only reduced respiratory compliance, but also increased resistance and ventilation heterogeneity. In the second stage of the study, correlation analysis revealed that abnormities in resistance, compliance, ventilation heterogeneity, and respiratory work were associated with the curvature of the spine.

Oscillometry requires only tidal breathing, is easy to perform, and provides a detailed analysis. These practical considerations, together with the present study's results, indicate that this technique may make a significant clinical contribution, representing an alternative and/or complement to spirometry in scoliosis.

## Supplementary Material

Click here to view [pdf].
